# Proteomics of appetite-regulating system influenced by menstrual cycle and intensive exercise in female athletes: a pilot study

**DOI:** 10.1038/s41598-024-54572-1

**Published:** 2024-02-20

**Authors:** Kazuhiro Tanabe, Kayoko Kamemoto, Yoshimasa Kawaguchi, Kai Fushimi, Sing Ying Wong, Nodoka Ikegami, Mikako Sakamaki-Sunaga, Nobuhiro Hayashi

**Affiliations:** 1 School of Life Science and Technology, Tokyo Institute of Technology, Ookayama, Meguro-ku, Tokyo Japan; 2 Medical Solution Promotion Department, Medical Solution Segment, LSI Medience Corporation, Shimura, Itabashi-ku, Tokyo Japan; 3 Graduate School of Physical Education, Health and Sport Science, Nippon Sport Science University, Fukasawa, Setagaya-ku, Tokyo Japan; 4 Department of Exercise Physiology, Nippon Sport Science University, Fukasawa, Setagaya-ku, Tokyo Japan

**Keywords:** Proteomics, Biomarkers, Health care

## Abstract

Female athletes who endure intense training are at risk of developing the 'female athlete triad,' making energy intake management crucial. However, the fluctuations in estradiol and progesterone levels throughout the menstrual cycle present a challenge in maintaining consistent energy intake. This study aimed to uncover the underlying factors associated with appetite regulation linked to menstrual phases and exercise using proteomic approach. Five female athletes engaged in 60 min of bicycle exercise, followed by 90 min of rest, during both the follicular and luteal phases. Serum samples were collected before, during, and after exercise, and the serum proteome was analyzed using 2D-gel electrophoresis. A total of 511 spots were detected in the subjects' serum profiles, with significant decreases observed in haptoglobin during the luteal phase and complement component 3 during bicycle training. Unsupervised learning with a generalized estimating equation analysis showed that serum peptide YY (PYY), an appetite suppressor, significantly influenced the fluctuations of serum proteins induced by exercise (*p* < 0.05). Regression analysis demonstrated a positive correlation between PYY and serum IgM (*R* = 0.87), implying that the intestinal environment and the immune response in female athletes may contribute to appetite regulation.

## Introduction

Management of the physical condition of female athletes is an essential issue, not only to maximize physical performance for athletic competitions, but also to prevent the occurrence of the “female athlete triad”: eating disorders (ED), menstrual dysfunction, and osteoporosis^1,2^. Ovarian hormones, such as estradiol and progesterone, play a critical role in regulating various aspects of female physiology, including energy intake and appetite. Estradiol directly stimulates anorexigenic neurons in the hypothalamus, reducing energy intake^3^, and also increases the secretion of cholecystokinin, a gut peptide hormone that stimulates anorexigenic neurons in the central nervous system, resulting in reducing appetite^4–7^. In contrast, progesterone suppresses the activity of anorexigenic neurons and stimulates the activity of orexigenic neurons, resulting in increasing energy intake^8^. It is worth noting that these two hormones tend to increase simultaneously during the luteal phase (following ovulation), and the complex interplay of their effects could have a notable impact on women's overall well-being. Disruptions in hormonal balance have been associated with various eating disorder^9–11^. Numerous studies have been conducted to elucidate the pathogenesis of ED, particularly involvement of “appetite-regulating hormones”, such as peptide YY (PYY) and acylated-ghrelin^12,13^. PYY is involved in energy intake through gastrointestinal digestion^14,15^, where it inhibits the orexigenic neuropeptide Y neurons in the hypothalamus as part of its central anorexigenic function^14^. On the other hand, acylated ghrelin is an orexigenic gut hormone that stimulates energy intake^16,17^ through the activation of neuropeptide Y neurons in the hypothalamus^14^. Galmiche et al.^12^ revealed the relationship between appetite-regulating hormones and ED; the concentrations of PYY were much higher in compulsive ED patients than in restrictive ED patients, suggesting resistance to the peptide in compulsive ED patients, and binge eating characterized by massive ingestion of high-fat and high-sugar food may stimulate an increased release of PYY. On the other hands, acylated ghrelin was higher in restrictive ED patients, suggesting that the limitation of energy intake may elicit increased ghrelin secretion.

Apart from ED patients, many past studies have also involved healthy women as research subjects to clarify the relationship between exercise, energy intake, and appetite-regulating hormones^18–20^. Douglas et al. recruited both lean and overweight/obese women and obtained data on appetite, energy intake, and appetite-regulating hormones during moderate-intensity exercise. They revealed that exercise transiently suppressed appetite by increasing PYY in both lean and overweight/obese individuals^18^. These results suggested that appetite-regulating hormones, such as PYY and acylated ghrelin, play an important role in controlling the appetite or energy intake of female athletes. However, many scientists have been perplexed by inconsistent findings, partly due to the fact that appetite-regulating hormones can be influenced not only by exercise but also by the menstrual cycle. Kamemoto et al. stressed the importance of matching the timing of menstrual phases with exercise bouts to obtain reliable outcomes. They recruited 10 female athletes belonging to a college softball team where they participated in two bicycle training sessions in the mid-luteal and the follicular phases (a period between menstruation and ovulation). Serum samples were collected five times (just before, during, immediately after, 45 min after, and 90 min after exercise), and ovarian hormones and appetite-regulating hormones were measured and analyzed^21^. Results demonstrated that exercise significantly increased estradiol and progesterone levels more in the luteal phase than in the follicular phase. However, no differences were observed in appetite-regulatory hormones, subjective appetite, or energy intake between the follicular and luteal phases. These altogether suggested that exercise-induced increases in ovarian hormones in the luteal phase may not influence appetite-regulating hormones in physically active women. Moniz et al*.* conducted a similar trial to investigate the impact of menstrual cycle and exercise on appetite-regulating hormones. They collected blood samples before, during, and after running exercise in both the follicular and luteal phases. The key distinction from Kamemoto's study was that participants consumed a meal just before the exercise. Their findings showed that acylated-ghrelin levels were lower in the follicular phase compared to the luteal phase. However, no differences were observed in PYY, GLP-1, and overall appetite between the follicular and luteal phases, while energy intake was greater in the luteal phase than in the follicular phase^22^.

Despite these existing researches, the precise mechanisms by which these hormones influence appetite regulation are still not fully understood. Therefore, more detailed exploration is needed to shed light on the specific functions and mechanisms of these hormones in relation to appetite control, particularly during physical exercise and across menstrual phases.

We hypothesized that there may be other underlying factors that are intricately involved in appetite regulation. Furthermore, we postulate that non-targeted comprehensive approaches may hold a bigger promise in uncovering these underlying factors compared to the current approach that focuses solely on specific biomarkers. Omics approaches emerged in the 2000s and currently contribute to various research areas. Proteomics is a widely-used approach for elucidating complicated protein functions, and 2D gel electrophoresis has been advantageous for detecting natural-form proteins, especially when targeting post-translational modifications^23–25^.

We aim to uncover the latent factors in female athletes' serum proteins that play a significant role in modulating appetite in response to the menstrual phases and intensive exercise.

## Material and methods

### Study design and recruiting participants

**Figure 1:**
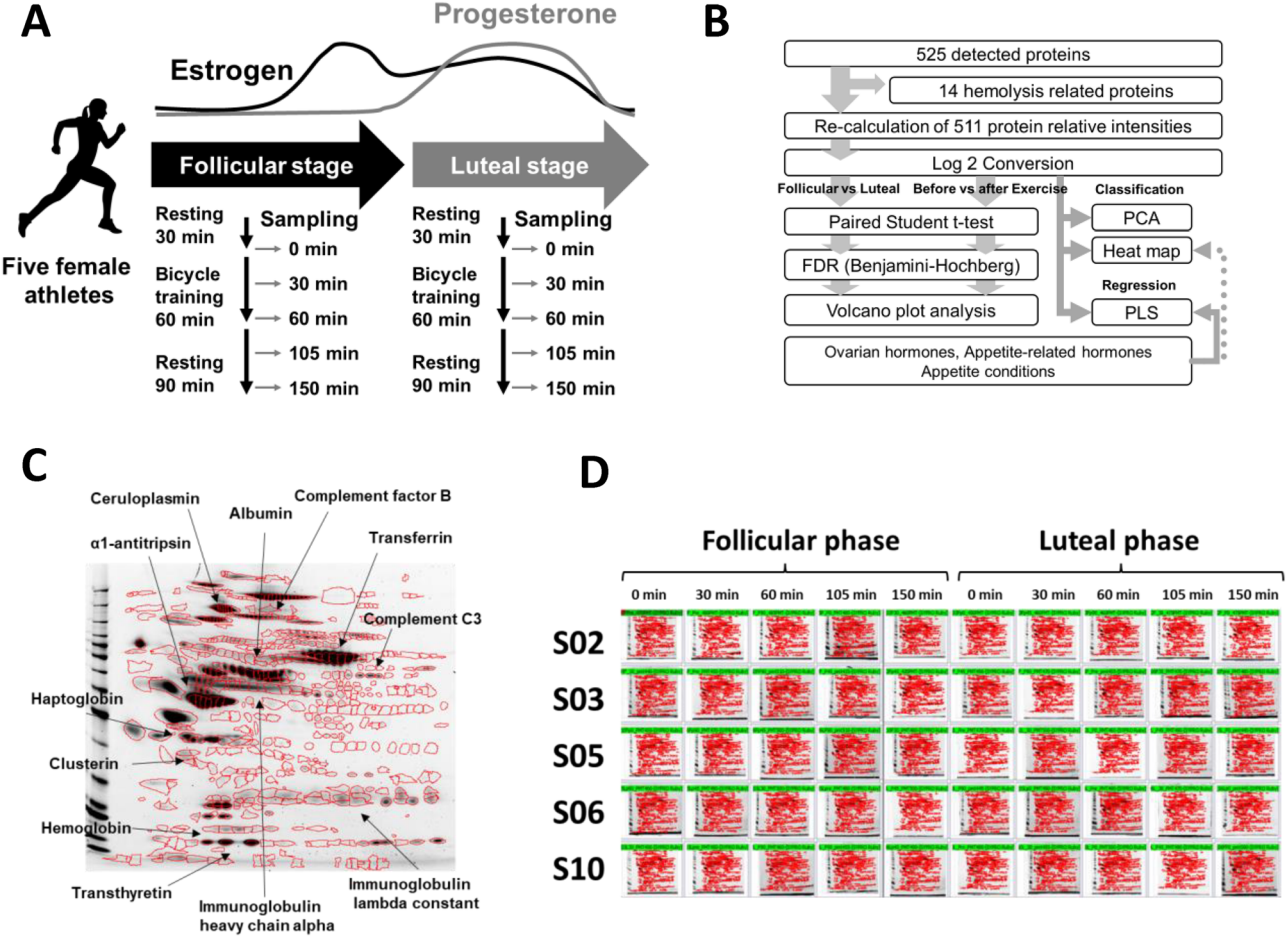
Study design and overview of serum protein expressions. (**A**) Sera were collected from five female students, just before, during, immediately after, 45 min after, and 90 min after exercise. This trial was repeated in both the follicular and luteal phases. (**B**) A typical 2D gel electrophoresis image of human serum. (**C**) An overview of 50 images (5 students × 2 phases × 5 sampling points) of 2D gel-electrophoresis. (**D**) Data mining process: After excluding erythrocyte-related proteins, the relative abundance ratio of the total 511 protein levels was summed up to 100%. Paired Student *t*-tests were performed between the luteal and follicular phases, as well as between pre- and post-exercise time points. The resulting *p*-values were adjusted for FDR using the Benjamini–Hochberg method. (**E**) PCA grouped by menstrual phases. (**F**) PCA grouped by exercise timing. (**G**) PCA grouped by individuals.

Fifteen female students belonging to a softball team were recruited who included non-smoking habits, absence of special diets, non-pregnancy, lack of diseases or medication intake, and no use of oral contraceptives and dietary supplements. The participants were trained 5 h/day and 6 days/week, and the trials conducted in the competitive season. Five participants were excluded due to irregular menstruation (a regular menstrual cycle was defined as having a consistent length between 25 and 38 days)^26^ or resigning from the team as a player^21^. Remining ten participants (age 20.6 ± 0.7 year; height 159.2 ± 2.8 cm) of body mass and body mass index (BMI) were 59.8 ± 7.9 kg; 23.6 ± 2.9 kg/m^2^ in the follicular phase, and 59.6 ± 7.6 kg; 23.5 ± 2.7 kg/m^2^ in the luteal phase, respectively. Participants arrived at the laboratory at 08:30 AM or 09:30 AM after a minimum of 10-h overnight fast (i.e., no food or drink except water), rested for 30 min, and then engaged in bicycle exercise at 70% of heart rate reserve [Target heart rate = (maximum heart rate − resting heart rate) × 0.7 + resting heart rate] for 60 min, followed by 90 min rest period. To assess potential differences in hormone levels, this experiment was conducted during both the follicular phases (defined as between 1 and 5 days from the first menstruation day) and luteal phases (defined as between 3 and 7 days before a menstruation day) of the menstrual cycle. Blood samples were collected at various intervals, including before exercise (0 min), during exercise (30 min), after exercise (60 min), during rest (105 min), and after rest (150 min) as illustrated in Fig. 1A. Blood samples were kept at room temperature (20–22 °C) for 30 min and centrifuged at 3000 rpm for 10 min at 4 °C to isolate serum. Sera were stored at 5 °C until ovarian hormone analysis, and kept at − 80 °C until PYY, acylated-ghrelin, and 2DE analyses. Serum concentrations of ovarian hormones and appetite-regulating hormones, PYY and acylated-ghrelin, were analyzed at LSI Medience Corporation, a clinical testing contract laboratory located in Tokyo, Japan. The precision (intra-day reproducibility) of these assays was all less than 5%^21^. While there are two forms of PYY: PYY1-36 and PYY3-36, where the latter being more potent as an appetite-suppressing signal than the first^15^, we analyzed total PYY as a regulator of the digestion process and appetite control according to the previous study^27^. Appetite was assessed using a 100 mm visual analogue scale (VAS) to assess hunger and satiety 0 min, 30 min, 60 min, 105 min, and 150 min^28^. All participants consumed 350 mL of water during the trial. Participants recorded their dietary intake on three days (the day before the experimental day, the experimental day, and the day after the experimental day) during the follicular and luteal phases. A registered dietitian asked the participants to confirm the contents of their food records and calculated the nutritional value of the food.

From the ten participants, five were further selected based on the following criteria: estradiol levels below 50 pg/mL and progesterone levels below 0.5 ng/mL during the follicular phase, followed by subsequent increases in both hormone levels during the luteal phase. Approval from the Institutional Review Board (IRB) was obtained from both Nippon Sport Science University (No. 017-H096) and Tokyo Institute of Technology (No. 2018045), and all participating women provided informed consent. All research methods and procedures were conducted in strict accordance with the ethical principles outlined in the Declaration of Helsinki, as well as in full compliance with relevant institutional, national, and international guidelines.

### Pretreatment of serum for 2DE analysis

Pretreatment of serum for the removal of major proteins that would otherwise impede the analysis of minor proteins in 2DE was performed following previously described methods^24,25^. Briefly, 60 µL of serum was mixed with 180 µL of dilution buffer (Seppro IgY 14 protein kit, Sigma-Aldrich Co., St. Louis, MO, US) and filtered through a Spin-X Column (0.45 µm, Corning, New York, U.S.) at 10,000 rpm. The filtered solution was subjected to the Aurum Serum protein Mini kit (BIO RAD, Hercules, CA, US) to remove albumin and IgG, following the manufacturer's instructions. Subsequently, 100 µl of the Aurum-treated solution was added to 550 µl of Dilution Buffer and loaded onto Seppro IgY14 Spin Columns to eliminate 14 major serum proteins, as per the manufacturer’s protocol. Randomization of the samples during preparation was performed to prevent potential biases caused by column degradation. The depleted fraction obtained from the Seppro IgY step (100 µl) was further treated with 2D Clean Up kit (Cytiva, MA, US) to remove salts and lipids. The resulting protein pellets were then dissolved in 100 µl of DeStreak Rehydration Solution (Cytiva), and the total protein concentration was determined using the 2D Clean Up kit (Cytiva).

### 2D-Electrophoresis

The 2DE experiment was conducted following previously reported methods^24,25^. Briefly, the DeStreak Rehydration Solution containing 50 µg of proteins was absorbed into Immobiline DryStrips (7 cm, pH 3–10, Cytiva, MA, US) for 18 h at room temperature. Isoelectric focusing (IEF) was carried out on a Multiphor II system (Cytiva). After the run, the strips were equilibrated in equilibration buffer (6 M urea, 1.5 M tris-hydrochloric acid, pH 8.8; 30% v/v glycerol; 2% sodium dodecyl sulfate) containing dithiothreitol (DTT, 10 mg/mL, 3 mL) for 15 min, followed by equilibrating with the buffer containing IAA (25 mg/mL, 3 mL) for another 15 min. Second dimensional gel electrophoresis is then performed using NuPAGE 4–12% Bis–Tris ZOOM GEL (Thermo Fisher Scientific, MA, US) under constant voltage of 200 V for 50 min. After the second-dimension separation, the gels were immersed in fixing solution (50% Methanol, 7% Acetic acid, 100 mL) for 30 min, followed by staining with the fluorescent dye (SYPRO Ruby, 40 mL, Invitrogen, MA, US) at room temperature for 12 h. The gels are then washed with pure water, followed by destaining with solution (10% Methanol, 7% Acetic acid, 100 mL) for 30 min. We acquired fluorescent images from the stained gels using a Typhoon FLA 9500 scanner (Cytiva). Finally, the gel images were aligned and analyzed using Melanie (Cytiva) where the spots %vol were extracted, in accordance to the previously reported method^25^.

### Data analysis and statistics

Before analyzing the proteins that impact appetite regulation, we excluded hemolysis-related proteins to minimize their influence on the data. Hemolysis-related proteins can have a significant impact on other proteins since we analyze the expression levels relative to the total protein level. Visual assessment was used to determine hemolyzed serum samples: samples with a pink or red color were classified as “hemolyzed” (*n* = 20), while samples that appeared transparent or yellow were considered “non-hemolyzed” (*n* = 30). We compared the hemolysis and non-hemolysis groups using Student t-test to identify hemolysis-related proteins. Proteins with false discovery rate (FDR) < 0.1 were considered as hemolysis-related proteins. After we excluded hemolysis-related proteins from the data table, we recalculated relative abundances of each protein, then logarithmically transformed the percentages, and performed paired Student t-tests to compare the expression levels of the 511 proteins between the luteal and follicular phases, as well as between pre- and post-exercise time points. Paired Student t-tests provides accurate *p*-values compared to unpaired Student *t*-tests, particularly when comparing two samples from the same individuals, thereby reducing the influence of inter-individual variations. Subsequently, the *p*-values were adjusted for FDR using the Benjamini–Hochberg method (Fig. 1B). To supplement the evaluation of significant differences, we introduced Cohen’s effect size (Cohen’s d), calculated by dividing the difference of averages by the standard deviation. Additionally, to assess the correlation between the two factors, we calculated the coefficient of correlations using the Pearson method. Principal component analysis (PCA) and uniform manifold approximation and projection (UMAP) were conducted using MATLAB (ver. 2023a, MathWorks, MA, US). Partial least squares analysis (PLS) was performed using proprietary software developed based on the following references^29^. In this model, we considered female conditions, ovarian hormones, and appetite-related hormones as the objective (dependent) variables (Y), while the 511 proteins were treated as the independent variables (X). To assess the reliability and robustness of the PLS regression model, we performed permutation evaluation. This technique involves randomly permuting the values of the objective variable Y and then re-estimating the PLS model using these permuted values. By repeating this process multiple times and comparing the results to the original PLS model, we were able to assess the reliability (robustness) of the original model and identify any potential overfitting^29^.

In addition, we further developed a novel approach called “transitional pattern classification” to simplify the categorization. Instead of relying on sole-protein expressions, this approach classifies samples based on the patterns of protein transitions during exercise. Firstly, we utilized PCA to identify three orthogonal basic transition patterns, next, we calculated cosine values for each protein transition pattern against the three bases. These cosines were used for hierarchical clustering to classify the participants. To aid further comprehension, we assigned three colors (RGB) to the three bases to visualize the categorization. After we classified examinees into some groups by transitional pattern classification, we investigated the relation to appetite or appetite-related hormones using a generalized estimating equation analysis.

### Protein identification: in-gel tryptic digestion and LC–MS/MS analysis

From the result of statistical analysis, target protein spots were identified according to previously reported methods^25^. Briefly, we used the Ettan Spot Picker (Cytiva) to automatically cut the target spots. The excised gel plugs were reduced and alkylated using 100 mM DTT and 50 mM IAA, before they were digested by trypsin (Trypsin Gold, Mass Spectrometry Grade, Promega, WI, US) at 37 °C overnight. The peptide solution was desalted and concentrated using a C-tip (AMR, Tokyo, Japan), and dissolved in a solution of 0.1% trifluoroacetic acid, 2% acetonitrile in ultra-pure water. We then injected this solution into a liquid chromatograph (Thermo Fisher Scientific, MA, US) equipped with a C18 column (100 µm diameter × 150 nm length, packed with 3 µm particles), and analyzed it using a quadrupole orbital trap mass spectrometer (Q-Exactive, Thermo Fisher Scientific) with an electrospray ionization interface and NIMS Proteome Discoverer 2.0 software (Thermo Fisher Scientific). The mobile phase consisted of 0.1% formic acid in water (A) and 100% acetonitrile (B), and the peptides were eluted at a flow rate of 500 nL/min with a gradient of 5–45% B buffer over 20 min at 35 °C. We used MASCOT (Version 2.5.1, Matrix Science, United Kingdom) along with the Swiss-Prot database (Swiss-Prot 2019_11, 561,568 sequences; 201,997,950 residues) to identify protein candidates from MS/MS spectra. The data underwent additional filtering to exclude the following: (1) candidates with Score Mascot less than 100 or FDR > 0.01, indicating low accuracy, (2) potential contaminants from the experiments such as keratin, and (3) candidates with low reproducibility, only identified once in two trials.

### IgM and PYY binding assay

One of the results in the present study was the high correlation between PYY and immunoglobulin M. To clarify the relation between PYY and immunoglobulin M, which may affect appetite regulation in female athletes, we established a binding assay between IgM and PYY as follows. Standard PYY (2.32 nmol, Fujifilm Wako Pure Chemical, Tokyo, Japan) was dissolved in PBS (10 µL, pH 7.4), and mixed with 300 nmol of NHS-biotin (30 µL, Thermo Fisher Scientific), followed by mixing for 60 min at room temperature to generate biotinylated PYY (PYY+). We also prepared a negative control by reacting L-glycine with NHS-biotin instead of PYY (PYY−). Then, we added serum (1.0 µL) to the biotinylated PYY solution or the negative control and incubate it for 30 min at room temp. The solution was filtered by Amicon 30K filtration (Milipore, MA, US) by centrifuging at 12,000 rpm for 10 min. Trapped proteins were washed with PBS, recovered with 120 µL of PBS, and mixed with 50 µL of Dynabeads M-280 Streptavidin (prewashed with PBS, Invitrogen, MA, US). It was incubated for 30 min at room temperature, and IgM-PYY complex was removed using magnet. The supernatant containing non-binding IgM was diluted 60 folds and analyzed by ELISA (Human IgM ELISA Kit, Abcam, Cambridge, UK). The procedure was followed by manual provided by the manufacturer. To check the validity of the established binding assay, anti-human PYY rabbit antibody (HPA010973, Sigma-Aldrich, MO, US) and Rabbit IgG ELISA Kit (ab187400, Abcam) were used.

## Results

### Study design and overview of serum protein expressions

Sera were collected from five female students at various time points: just before exercise (0 min), during exercise (30 min), after exercise (60 min), during rest (105 min), and after rest (150 min).

This trial was repeated in both the follicular and luteal phases of the menstrual cycle (Fig. 1A). A total of 50 images (5 students × 2 phases × 5 sampling points) of 2D gel-electrophoresis were obtained, and a total of 525 protein spots were detected (Fig. 1C,D; All raw images are shown in Supplementary Fig. 1 and 2). When we checked the hemolysis based on visual subjective assessment, we observed 20 hemolyzed sera out of the 50 samples, and when Student *t*-test was performed between the 20 hemolyzed and 30 non-hemolyzed samples for each detected protein, 14 proteins with FDR < 0.1 were determined to be hemolysis-related proteins and were subsequently excluded from the data table.

### Impact of serum protein variations on menstrual cycle and bicycle exercise

**Figure 2:**
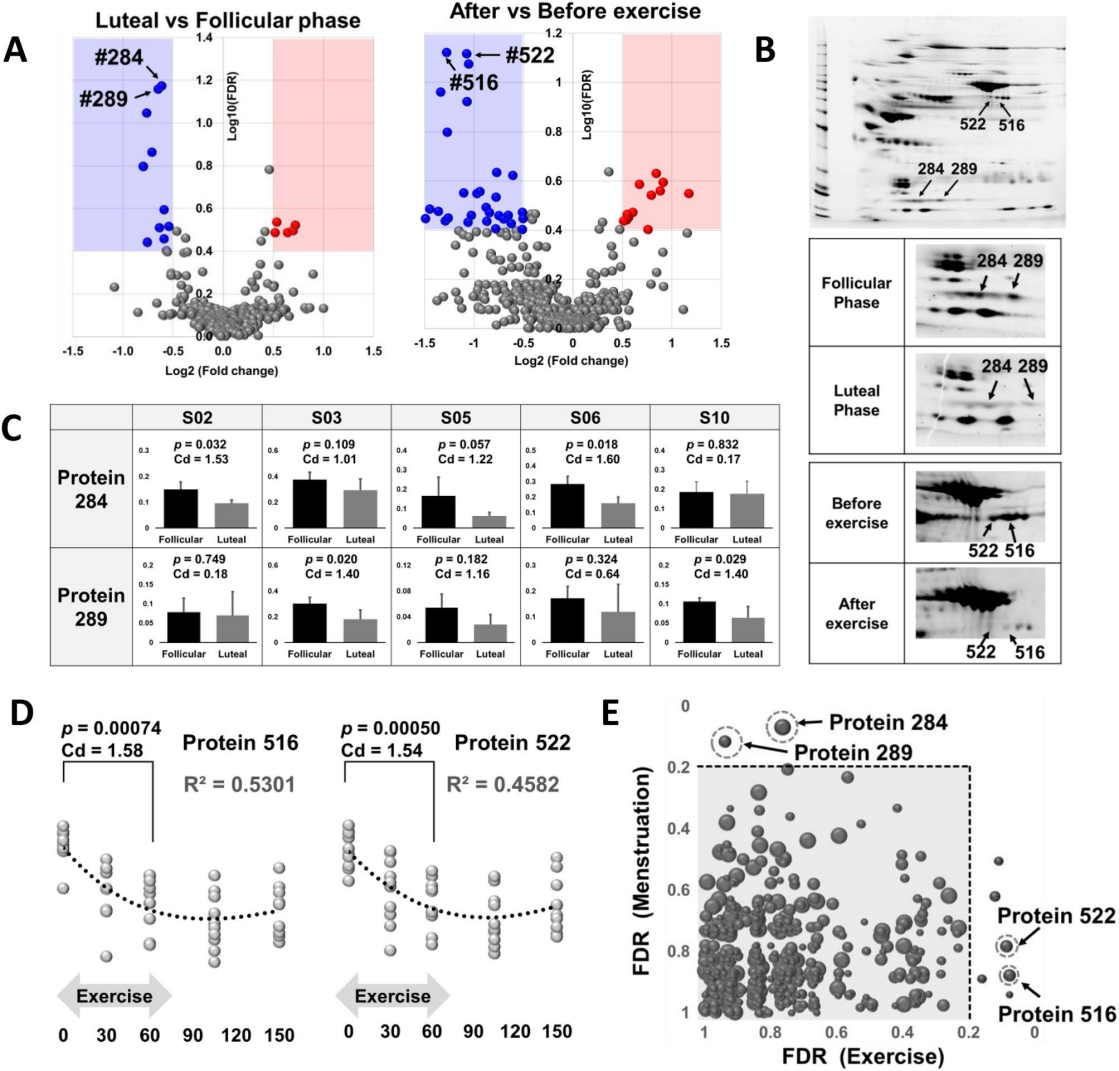
Impact of serum protein variations on menstrual cycle and bicycle exercise. (**A**) Volcano plot showing the comparison between the luteal phase and follicular phase (left) and the comparison between after exercise and before exercise (right). The horizontal axis represents Log2 (mean-fold-changes), the vertical axis represents Log10(FDR). (**B**) Spot positions of proteins #284, #289, #516, and #522 in 2DE. Upper panel: Visualization of spot positions on a 2D gel. Middle panel: Representative gel images of the follicular and luteal phases. Lower panel: Representative gel images before and after exercise. (**C**) Expression levels of protein #284 and #289 between the follicular and luteal phases in each individual. (**D**) Transition patterns of proteins #516 and #522 during bicycle exercise. (**E**) Scatter plot using the false discovery rates (FDRs) of the menstrual cycle (vertical axis) and exercise (horizontal axis). The size of each dot represents the expression levels. Cd: Cohen’s d.

Volcano plot analysis demonstrated a significant decrease in protein #284 and #289 during the luteal phase compared to the follicular phase (Fig. 2A, left). These proteins had an estimated molecular weight (MW) of 20 kDa and approximate isoelectric points (pI) of 5.2 (#284) and 5.9 (#289) on 2DE (Fig. 2B, upper), and LC–MS/MS identified these spots as haptoglobin (Table 1). Same identification results of #284 and #289 suggests that haptoglobin was post-translational modified and had different charges. Student t-test analysis observed significant decreases (*p* < 0.05) in protein #284 levels in S02 and S06, and those in protein #289 levels in S03 and S10 between the follicular and luteal phases. Cohen's effect size (Cohen's d) indicated substantial decreases (Cohen’s d > 1) in spot #284 for participants S02, S03, S05, and S06, and in spot #289 for participants S03, S05, and S10 between the follicular and luteal phases (Fig. 2C).

**Table 1:** Identification of the spots in 2DE-gel by LC–MS/MS.

# Spot	Accession	Description	Coverage (%)	# Peptides	# PSMs	# Unique peptides	# AAs	MW (kDa)	Calc. pI	Score mascot
284	P00738	Haptoglobin OS = Homo sapiens OX = 9606 GN = HP PE = 1 SV = 1	7	5	8	5	406	45.2	6.58	195
			7	3	3	3				113
289	P00738	Haptoglobin OS = Homo sapiens OX = 9606 GN = HP PE = 1 SV = 1	11	7	10	7	406	45.2	6.58	200
			7	4	4	4				118
516	P01024	Complement C3 OS = Homo sapiens OX = 9606 GN = C3 PE = 1 SV = 2	23	38	49	38	1663	187	6.4	1452
			21	36	55	36				1702
522	P01024	Complement C3 OS = Homo sapiens OX = 9606 GN = C3 PE = 1 SV = 2	16	30	37	30	1663	187	6.4	815
			27	40	64	40				2021
	P02787	Serotransferrin OS = Homo sapiens OX = 9606 GN = TF PE = 1 SV = 4	17	13	13	13	698	77	7.12	268
			28	17	18	17				391
800	P01871	Immunoglobulin heavy constant mu OS = Homo sapiens OX = 9606 GN = IGHM PE = 1 SV = 4	21	9	14	9	453	49.4	6.77	417
			21	9	14	9				417

The results of duplicated trials were described. #Spot: spot ID in 2DE. Accession: a unique identifier assigned to a specific protein in UniProt or NCBI. Description: an annotation of the identified protein. Coverage (%): percentage of the protein sequence that is covered by the identified peptides. #Peptides: the number of unique peptides identified for a particular protein. #PSMs: “Peptide Spectrum Matches.” The number of spectra (mass spectrometry data) that match to a specific peptide sequence. #Unique peptides: the number of distinct peptides that are uniquely assigned to a specific protein. #AAs: the number of amino acids in the protein sequence. MW (kDa): molecular weight. Calc. pI: calculated isoelectric point (pI) of the protein. Score Mascot: a score assigned by the MASCOT to evaluate the quality of the protein identification. #522 was also identified as serotransferrin; however, we concluded that serotransferrin was contaminated from its nearby original spot.

Volcano plot analysis also showed a significant decrease in protein #516 and #522 during bicycle exercise (comparison between 0 and 60 min, Fig. 2A, right). These proteins had an estimated molecular weight (MW) of 70 kDa and approximate isoelectric points (pI) of 7.0 (#516) and 6.9 (#522) on 2DE (Fig. 2B, upper), and LC–MS/MS identified these spots as complement component 3 (Table 1). Both #516 and #522 were considered as complement component 3 with different post-translational modifications. After they decreased during exercise (Fig. 2B, lower), these proteins exhibited a slight recovery during resting period (Fig. 2D). Additionally, we conducted a scatter plot analysis using false discovery rates (FDRs) to identify serum proteins affected by both the menstrual cycle and exercise. Result shows that few proteins were located in the top-right area of the plot (Fig. 2E), indicating a limited number of proteins that are responsive to both changes in menstrual cycle and exercise in the present study.

### Unsupervised learning to uncover underlying factors

**Figure 3:**
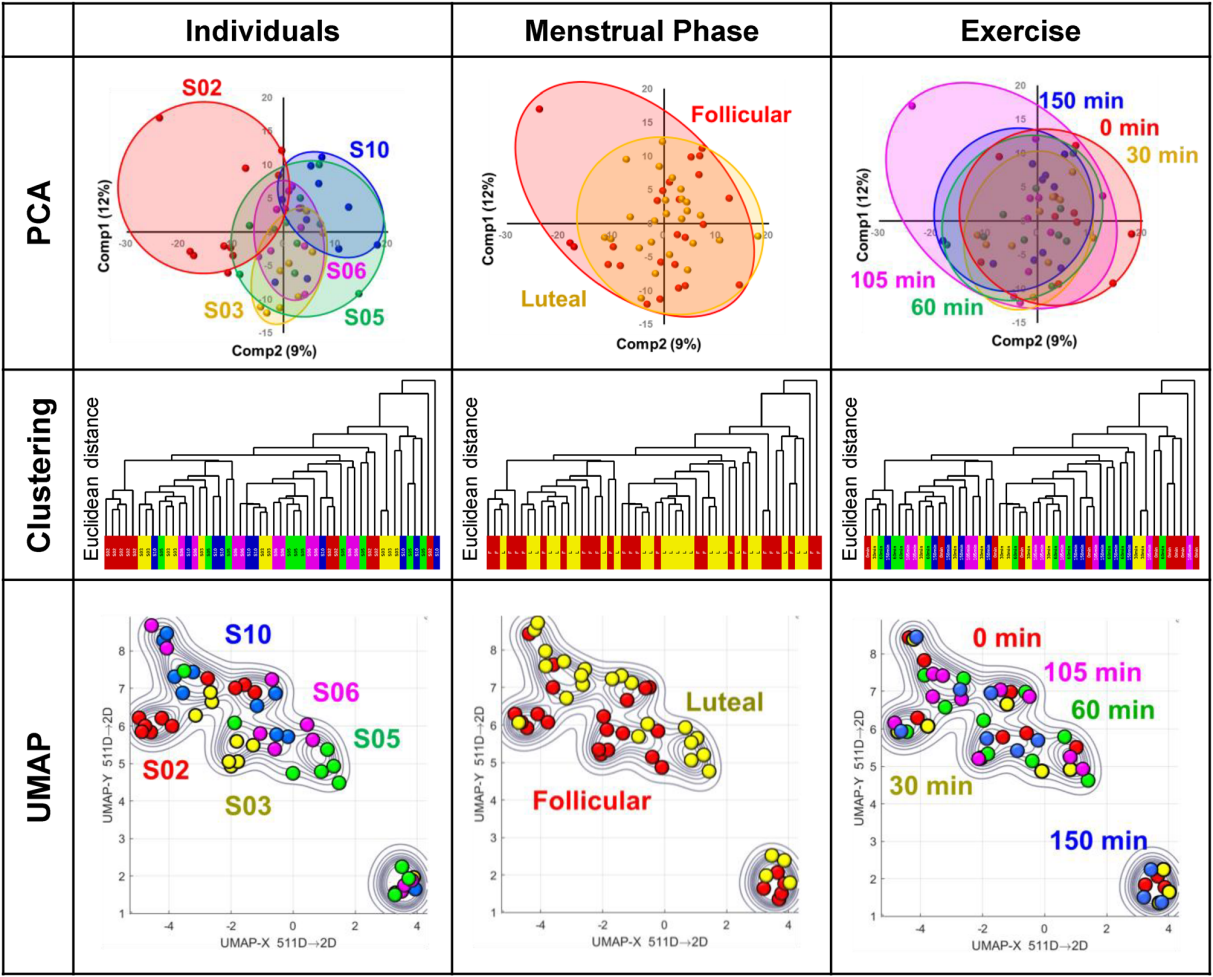
Principal component analysis (PCA), cluster analysis, and uniform manifold approximation and projection (UMAP). Unsupervised learning techniques, including PCA (upper), cluster analysis (middle), and UMAP (bottom), were employed to examine differences between individuals (left), menstrual phases (middle), and exercise states (right). The distance measure of the dendrogram is Euclidean distance. The color codes represent individuals: S02 (red), S03 (yellow), S05 (green), S06 (pink), and S10 (blue); menstrual phases: follicular phase (red) and luteal phase (yellow); and exercise periods: 0 min (red), 30 min (yellow), 60 min (green), 105 min (pink), and 150 min (blue).

We applied an unsupervised learning approach to uncover potential hidden factors in serum proteins that could impact the condition of female athletes. Although unsupervised learning has a drawback in that major variations can overshadow minor (yet important) variations, it has an advantage to provide unexpected clues to uncover underlying biological information. Principal component analysis (PCA) demonstrates a slight difference among the five individuals, particularly with the distribution of S02 appearing distinct from the others. However, no significant differences were observed between the follicular and luteal phases, or among the before, during, and after-exercise states (Fig. 3, upper). This suggests that individual variability in serum proteins is much larger than the changes associated with the menstrual cycle and exercise. In addition, a clustering analysis using a dendrogram was performed to supplement the PCA analysis. The distance measure is Euclidean distance. The results showed that the influence of individual differences was dominant as well as the PCA analysis. More notably, exercise also played a partial role in the grouping, with the 0-min sample in particular showing more separation than the other times (Fig. 3, middle).

Moreover, we utilized uniform manifold approximation and projection (UMAP), another dimensionality reduction method similar to PCA but employing a non-linear approach. UMAP offered a broad classification of individuals and highlighted subtle distinctions between the follicular and luteal phases. However, the alterations during exercise were less conspicuous as well as PCA (Fig. 3, bottom).

**Figure 4:**
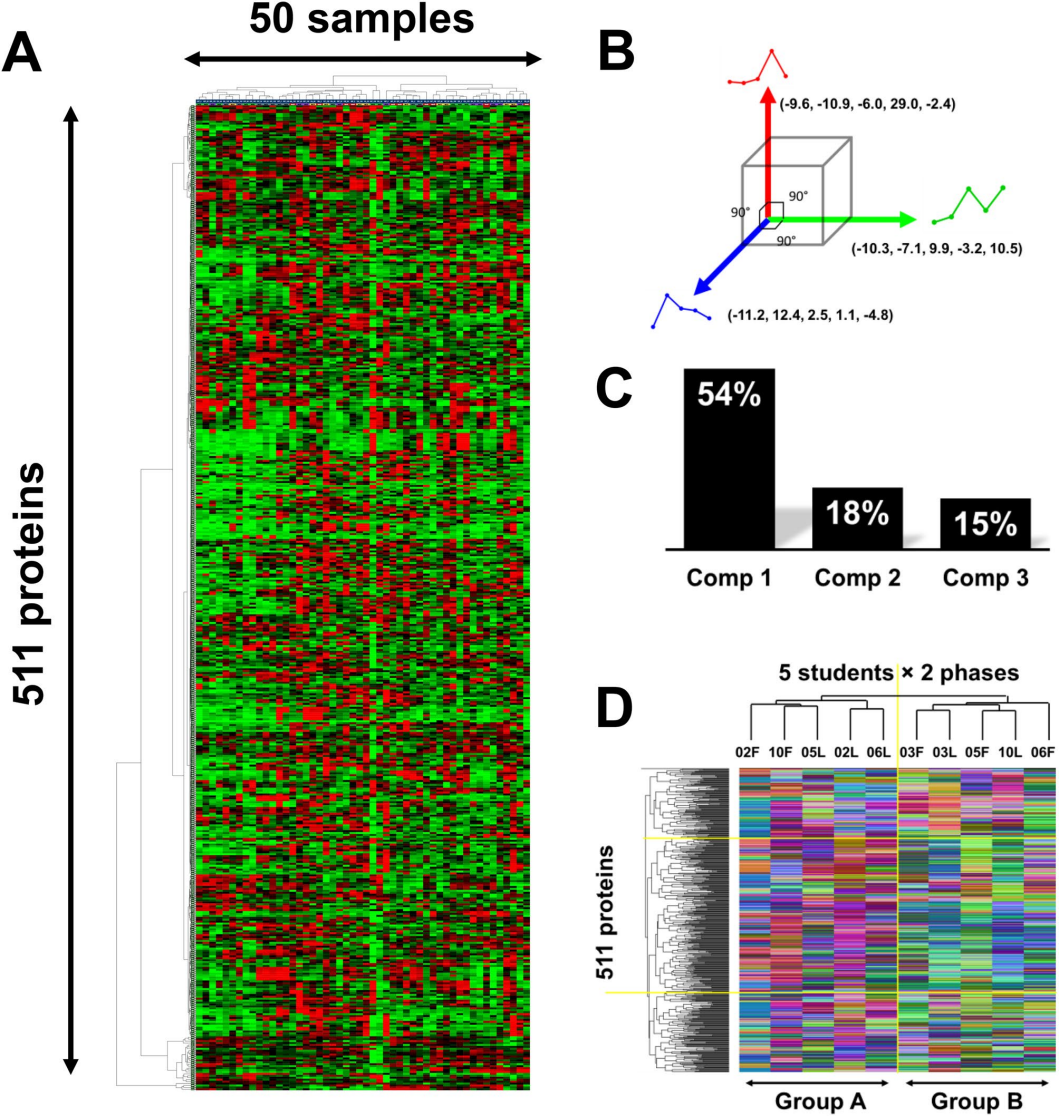
Heatmap analysis and transition pattern classification. (**A**) Heatmap analysis depicting the expression levels of 511 proteins across 50 samples: red: up-regulated, green: down-regulated. Proteins and samples were categorized by cluster analysis. (**B**) Three orthogonal transition bases obtained by PCA analysis. (**C**) Contributions of each principal components to the original data. (**D**) Transitional pattern heatmap with cluster analysis; Each protein transition pattern during exercise dissolved into three basic patterns, and the cosines to the three bases were used for categorization. Cosines were further converted to RGB colors to visualize the categorization.

Furthermore, a heatmap analysis was conducted to visualize the expression patterns of the 511 proteins across the 50 samples. Unfortunately, no obvious regularities emerged in the grouping, rendering it challenging to identify latent factors (Fig. 4A). We then applied a transitional pattern classification, where it generated three orthogonal basic transition patterns (Fig. 4B), each contribution to the original data was 54%, 18% and 15% respectively (Fig. 4C). One major advantage of transitional pattern classification, as opposed to the general hierarchical clustering analysis using all 50 samples, is its consideration of the temporal order of exercise time points. Interestingly, we observed that the transitional pattern classification assigned 10 samples roughly into two groups. One group included S02F, S10F, S05L, S02L, and S06L, while the other comprised S03F, S03L, S05F, S10L, and S06F (Fig. 4D). No clear pattern of categorization was seen for menstrual phases (luteal and follicular).

To further understand the potential factor of this classification, we performed a generalized estimating equation (GEE) analysis on the two separated groups. The analysis revealed significant differences in the subjective feeling of satiety^21^ (*p* = 0.044) and PYY levels (*p* = 0.028) between the two groups (Table 2). On the other hand, the other appetite-related factors, such as carbohydrate, lipid, protein or energy intake^21^, did not show any significant differences between the two groups. These findings suggest that PYY may be a key factor, which could play an important role associated to exercise.

**Table 2:** Generalized estimating equation (GEE) analysis.

Items	*P*-values
Estardiol	0.763
Progesterone	0.595
Acylated ghrelin	0.323
Total PYY	0.028
CCK	0.261
Hungry	0.352
Satiety	0.044
Energy intake	0.892
Protein intake	0.948
Lipid intake	0.346
Carbohydrate intake	0.45

*P*-values calculated using the generalized estimating equation method, which contributed to the separation between Group A and Group B.

### Regression analysis between appetite-regulating hormones and serum proteome

**Figure 5:**
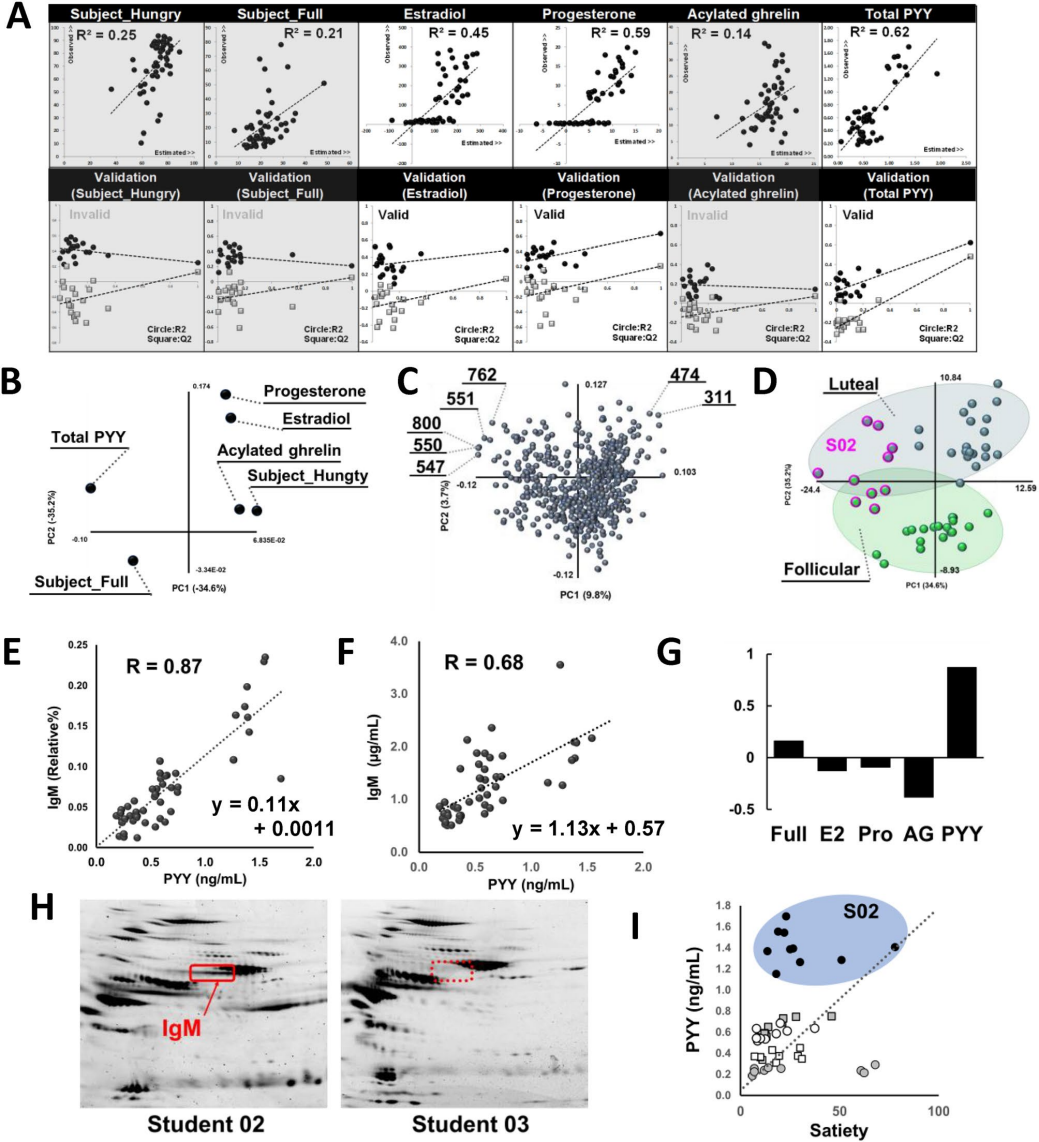
Regression analysis between appetite-regulating hormones and serum proteome. (**A**) Permutation evaluation of PLS; It generates randomly permuting the values of the objective variable Y and re-estimating PLS model using the permuted Y values. By repeating this process many times and comparing the results to the original PLS model. (**B**) PLS loading plot of objective variables Y. (**C**) PLS loading plot of independent variables X. (**D**) PLS score plot. (**E**) Correlation between PYY and IgM analyzed by 2DE. (**F**) Correlation between PYY and IgM analyzed by ELISA. (**G**) Coefficient of correlation between IgM analyzed by 2DE and objective factors. (**H**) Comparison of gel images between S02 and S03. (I) Correlation between self-repot satiety and PYY.

Based on the insights gained from the transitional pattern classification, we further explored the relationship between appetite-regulating hormones and serum proteins affected by exercise and the menstrual cycle. The primary goal of the PLS analysis was to investigate the relationships between linear combinations of the protein variables (X) and those of the hormone variables (Y) and identify any latent connections. As a result of the PLS analysis, the permutation evaluation indicated that ovarian hormones (estradiol and progesterone) and PYY exhibited significant correlations with the 511 serum proteins, while other factors such as hunger, fullness, or acetyl-ghrelin did not show distinct correlations (Fig. 5A). Upon comparing the PLS loadings of the objective variables (Fig. 5B), loadings of the independent variables (Fig. 5C), and the scores (Fig. 5D), it was obvious that PYY demonstrated a strong correlation with protein #800, and these factors were highly expressed in S02, as all of them were located in the left-upper region of the maps. Spot #800 (protein #800), was subjected to in-gel digestion using trypsin and analyzed through LC–MS/MS, which identified it as immunoglobulin M (IgM) (Table 1). IgM (Spot #800) exhibited a high correlation (*R* = 0.87, Fig. 5E) with PYY, and the serum concentrations quantified by ELISA also displayed a positive correlation (*R* = 0.68) with serum PYY (Fig. 5F). Conversely, other factors such as subjective fullness, estradiol, progesterone, or acetyl-ghrelin did not demonstrate significant correlations with IgM (Fig. 5G). The serum level of IgM remained stable throughout the menstrual cycle and did not show any significant changes even during exercise (Supplementary Fig. 3).

IgM showed particularly strong expression in S02 compared to the other students (Fig. 5H). When plotting the 50 samples based on PYY levels and subject satiety, it became apparent that S02 did not experience satiety despite having a high level of PYY (Fig. 5I). When examining the effects of appetite-related factors (subject hunger and satiety, appetite-regulating hormones) or ovarian hormones on haptoglobin and complement component 3, we did not observe any significant correlations between them (Supplementary Figs. 4, 5 and 6). These findings indicate that the changes in these serum proteins may not be directly involved in appetite-related factors.

### Development of binding assay system for IgM and PYY

**Figure 6:**
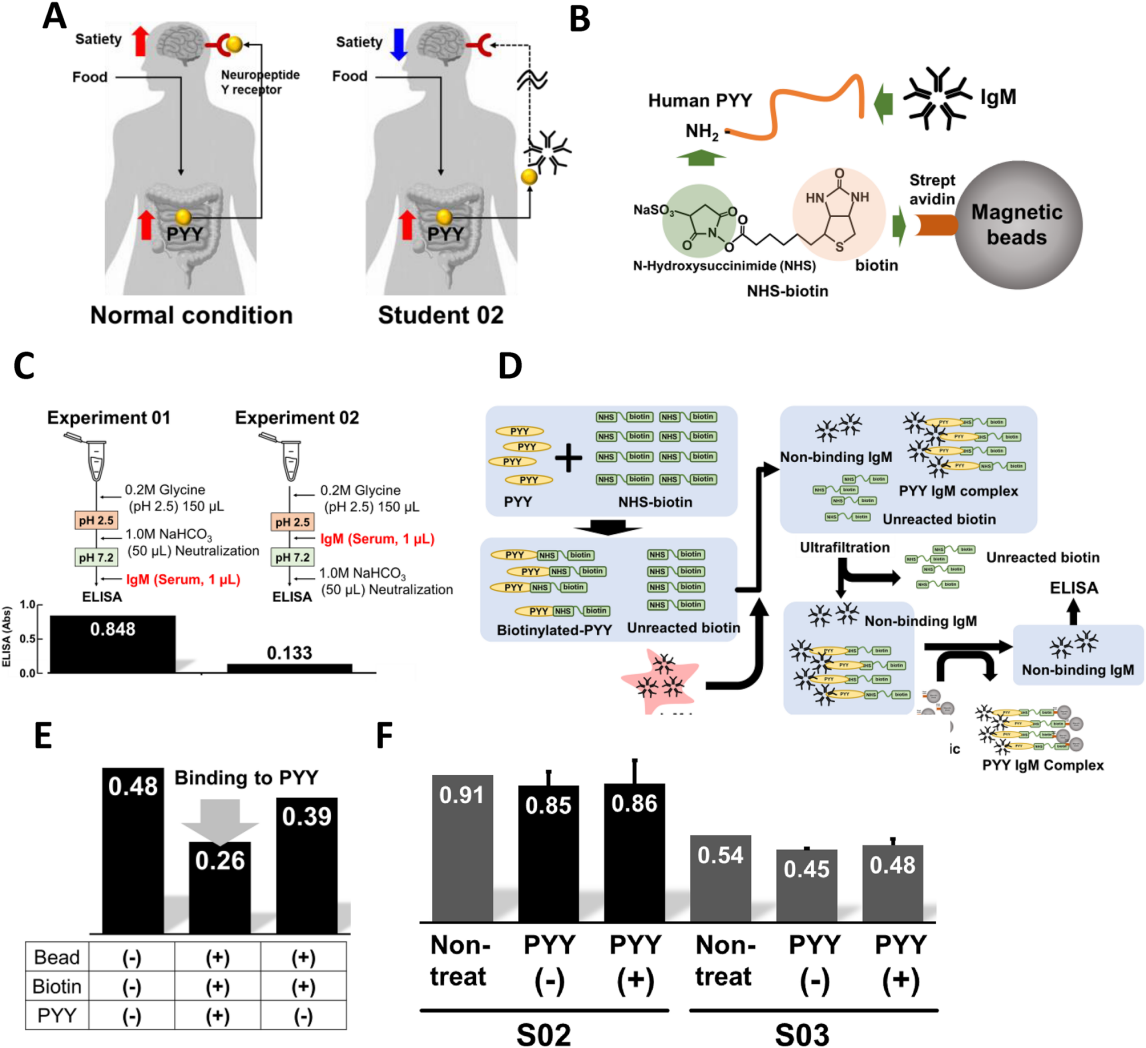
Hypothesis of IgM function and binding assay. (**A**) Hypothesis: IgM in S02 may be an autoantibody to PYY that impairs the anorexigenic function of PYY. (**B**) An image of binding assay to analyze the direct binding activity between PYY and IgM. (**C**)  Influence of acid treatment of IgM for ELISA analysis (**D**) Strategy and flow of the binding assay: excess biotin-tag with N-hydroxysuccinimide (NHS) was added to PYY standard to create a biotinylated PYY. Then, serum was added to the biotinylated PYY to form an IgM-PYY-biotin complex. Unreacted biotin-tag was removed using a 30K ultrafiltration, and the IgM-PYY complex was further separated using a magnet. Free IgM in the supernatant was analyzed using an ELISA kit. The amount of IgM bound to PYY was estimated by subtracting the signal of PYY(+) from that of PYY(−), which was prepared by reacting glycine with NHS instead of PYY. (**E**) Evaluation of the binding assay using anti-human PYY rabbit IgG as a positive control. (**F**) The amount of non-binding IgM of S02 and S03.

Based on the observations that S02 had high levels of PYY in the serum despite being in a fasting state, did not experience satiety despite the elevated PYY levels, and showed significantly elevated IgM expression (which correlated strongly with PYY), we hypothesized that IgM in S02 may act as an autoantibody to PYY, potentially interfering with the anorexigenic function of PYY (Fig. 6A). Previous studies have reported the presence of autoantibodies targeting appetite-regulating hormones, which can disrupt appetite balance^30–36^. To test this hypothesis, we established an assay system to detect direct binding of PYY to serum IgM (Fig. 6B). In this system, we quantified the non-binding fraction of IgM to avoid IgM denaturing in an acidic environment during the detachment process from PYY, which disabled ELISA detection (Fig. 6C). We added an excess amount of NHS-biotin to PYY, followed by adding serum IgM before introducing streptavidin-coated magnetic beads. Since the biotinylated PYY generates IgM-PYY-biotin complex, unreacted NHS-biotin can be easily removed using ultrafiltration (30 k Da). After removing the IgM-PYY-biotin complex using streptavidin magnetic beads, the amount of free (non-binding) IgM was analyzed using an ELISA kit (Fig. 6D). To validate the assay's reliability, we used anti-human PYY rabbit IgG as a positive control, which showed a clear decrease in binding to PYY(+) beads compared to PYY(−) beads (Fig. 6E). However, when we applied S02 serum to this assay, we did not observe any differences between PYY(+) and PYY(−), suggesting that IgM in S02 was not an autoantibody against PYY, which does not bind to PYY directly (Fig. 6F).

## Discussion

Female athletes engaged in intense activities such as gymnastics, ballet, or long-distance running face an increased risk of developing the 'female athlete triad', which includes eating disorders. This condition often arises due to low energy availability, wherein athletes struggle to consume sufficient energy to match the demands of their training. To gain insight into the intricate mechanisms involved in energy intake regulation, we specifically investigated appetite regulation and explored potential clues within serum proteins.

Upon investigating protein alterations between the follicular and luteal phases of the menstrual cycle, we observed a significant decrease in haptoglobin levels during the luteal phase, indicating an increase in its levels during the follicular phase. Haptoglobin is a protein known to bind to free hemoglobin released from erythrocytes, and its levels are associated with the loss of complement blood hemoglobin during menstruation^37^, therefore, it is reasonable to observe the increase in haptoglobin levels during the follicular phase, reflecting the normal physiological response. Furthermore, we observed a significant decrease in the serum level of complement component 3 during exercise. Complement component 3 plays a crucial role in the immune system's complement system. Kostrzewa-Nowak et al. demonstrated that intense exercise leads to a decrease in post-exercise C3 levels^38^, suggesting that the exercise may activate the classical pathway of complement activation while selectively downregulating C3 synthesis^39^. This exercise-induced reduction in complement component 3 has also been observed in animal models^40^. Intense exercise can sometimes lead to muscle disruption, resulting in an increase in serum myoglobin^41^. However, in this study, there was no observed elevation of muscle-related proteins in serum.

During our investigation of the correlation between these proteins and menstrual phase or exercise, we did not find any significant associations. As a result, the observed changes in both haptoglobin and complement component 3 appear to be typical responses to the menstrual cycle and exercise, respectively, and may not have a direct impact on appetite regulation.

To uncover latent factors that impact on appetite control caused by menstrual cycle, we introduced several statistical tools in this study. Unsupervised learning technique, such as PCA, UMAP, clustering analysis provided valuable information to overview the sample distribution, however, they were less efficient in uncovering latent factors. In contrast, the transitional pattern classification and the generalized estimating equation analysis revealed that PYY may play a crucial role in exercise-induced protein changes. Furthermore, the regression model using PLS revealed a strong association between serum IgM levels and PYY. This finding led us to hypothesize that IgM may act as an autoantibody to modulate the anorexic function of PYY. This hypothesis finds supports in several previous studies^30–36^. To validate our hypothesis, we have developed an assay system to quantify the direct binding between IgM and PYY: (1) quantifying the amount of binding IgM by subtracting the non-binding IgM fraction from the total IgM level, and (2) combining IgM (serum) with biotinylated PYY, removing unreacted NHS-biotin using ultrafiltration, and then adding streptavidin-coated beads to remove IgM binding to PYY. However, the results did not show any direct binding between IgM and PYY, which prompted us to explore alternative possibilities regarding the correlation between IgM and PYY. In a related study, Magri et al. investigated secretory IgM produced by plasma cells, a type of B cell, located in the gut mucosa. They found that gut IgM exhibited a wide range of targets among commensal bacteria, with a particularly strong response to *Firmicutes*^42^. This discovery provided us with insights to unravel the underlying connection between IgM and PYY. Considering that PYY secretion is triggered by short-chain fatty acids (SCFA) produced by the gut microbiome^43–45^, and *Firmicutes* are known to be more efficient in generating SCFA compared to other gut microbiome^46^, the proliferation of *Firmicutes* in the gut may serve as a stimulant for SCFA production, leading to increased PYY secretion and concurrent stimulation of plasma cells to produce IgM.

On the contrary, we should not disregard the hypothesis that IgM acts as an autoantibody against appetite-related hormones. Following the identification of IgM autoantibodies against alpha-melanocyte-stimulating hormone (α-MSH) in patients with anorexia nervosa by Fetissov et al.^32^, several studies have been conducted to investigate the involvement of IgM autoantibodies in anorexia nervosa. Roubalova et al.^47^ found decreased levels of α-MSH and elevated levels of anti-α-MSH IgM in patients with anorexia nervosa, suggesting that the interaction between the immune system and gut bacterial diversity plays a role in the pathogenesis of anorexia nervosa. Although we did not analyze α-MSH in this study, the possibility that IgM acts as an autoantibody against α-MSH remains plausible. Given that α-MSH inhibits the production of neuropeptide Y as well as PYY, it is not surprising that the levels of PYY and α-MSH are synchronously changed.

Finally, we acknowledge the limitations of this study. While we standardized participants' conditions, such as diet, menstrual cycle, rest periods, and exercise load, the intervention to personal lives had limitations. Efforts to minimize bias were undertaken, but certain risks must be considered. Firstly, the sample size was small, consisting of only 50 samples from five female participants, which may be considered insufficient for the study targeting complex mechanisms such as appetite regulation induced by menstrual cycle and exercise, as these factors can be strongly influenced by individual differences. Further research with a larger sample size is required to uncover latent factors impacting on appetite controls in female athletes. Assuming that the average concentration of immunoglobulin M would differ by no more than half of the standard deviations within each group, ideal sample size would be more than 100, considering an alpha-error (0.05) and beta-error (0.2) framework. Additionally, this study did not include female athletes with health issues, specifically those with eating disorders, which limited the exploration of potential relationships between eating disorders and variations in serum proteins. In future studies, it would be beneficial to collect not only serum samples but also fecal samples from each participant to gain a deeper insight into the potential mechanisms underlying eating disorders and their interactions with the gut microbiome. The integration of metagenome sequencing, such as 16S rRNA analysis, could offer valuable insights and elucidate the intricate connections between specific gut bacteria, SCFA production, and the secretion of appetite-regulating hormones like PYY^48^. This comprehensive approach would enable us to unravel the complex interplay between the host's physiological responses, gut microbial communities, and eating disorders.

## Conclusions

As findings that will lead to some molecular understanding of the relationship between menstrual timing and the effects of exercise, we have observed significant decreases in haptoglobin during the luteal phase and in complement component 3 during exercise. However, these changes did not show any correlations with appetite-related factors. On the other hand, our analyses using transitional pattern classification and generalized estimating equation have revealed that serum PYY levels significantly influenced the fluctuations of serum proteins induced by exercise (*p* < 0.05). Additionally, our regression analysis (PLS) indicated a positive correlation between PYY and serum IgM (*R* = 0.87), but no direct interactions at the molecular level were detected. In light of the profound impact of gut microbiome on energy intake, weight gain, and the immune system, maintaining the balance of the gut microbiome could play a crucial role in preventing the female athlete triad. In this context, both serum PYY and IgM, known for their strong associations with the gut microbiome, could serve as novel indicators for monitoring the overall condition of female athletes.

### Supplementary Information

Supplementary Figure 1.

Supplementary Figure 2.

Supplementary Figure 3.

Supplementary Figure 4.

Supplementary Figure 5.

Supplementary Figure 6.

Supplementary Legends.

Supplementary Information 8.

## Data Availability

All raw data we used in this study can be accessed in Supplementary Material.

## Abbreviations

2DE: Two-dimensional electrophoresis
PCA: Principal component analysis
UMAP: Uniform manifold approximation and projection
PLS: Partial least squares
FDR: False discovery rate
ED: Eating-disorders
PYY: Peptide YY
ELISA: Enzyme-linked immune-sorbent assay
IgM: Immunoglobulin M
IgG: Immunoglobulin G
IAA: Iodoacetamide
DTT: Dithiothreitol
LC–MS/MS: Liquid chromatography mass spectrometry
MW: Molecular weight
SCFA: Short-chain fatty acids
