# Publisher Correction: Proteomics of appetite-regulating system influenced by menstrual cycle and intensive exercise in female athletes: a pilot study

**DOI:** 10.1038/s41598-024-57180-1

**Published:** 2024-03-21

**Authors:** Kazuhiro Tanabe, Kayoko Kamemoto, Yoshimasa Kawaguchi, Kai Fushimi, Sing Ying Wong, Nodoka Ikegami, Mikako Sakamaki-Sunaga, Nobuhiro Hayashi

**Affiliations:** 1 School of Life Science and Technology, Tokyo Institute of Technology, Ookayama, Meguro-ku, Tokyo Japan; 2 Medical Solution Promotion Department, Medical Solution Segment, LSI Medience Corporation, Shimura, Itabashi-ku, Tokyo Japan; 3 Graduate School of Physical Education, Health and Sport Science, Nippon Sport Science University, Fukasawa, Setagaya-ku, Tokyo Japan; 4 Department of Exercise Physiology, Nippon Sport Science University, Fukasawa, Setagaya-ku, Tokyo Japan

Correction to: *Scientific Reports* 10.1038/s41598-024-54572-1, published online 20 February 2024

In the original version of this Article a previous rendition of Figure 4 was published. The original Fig. 4 and accompanying legend appear below.

**Figure 4:**
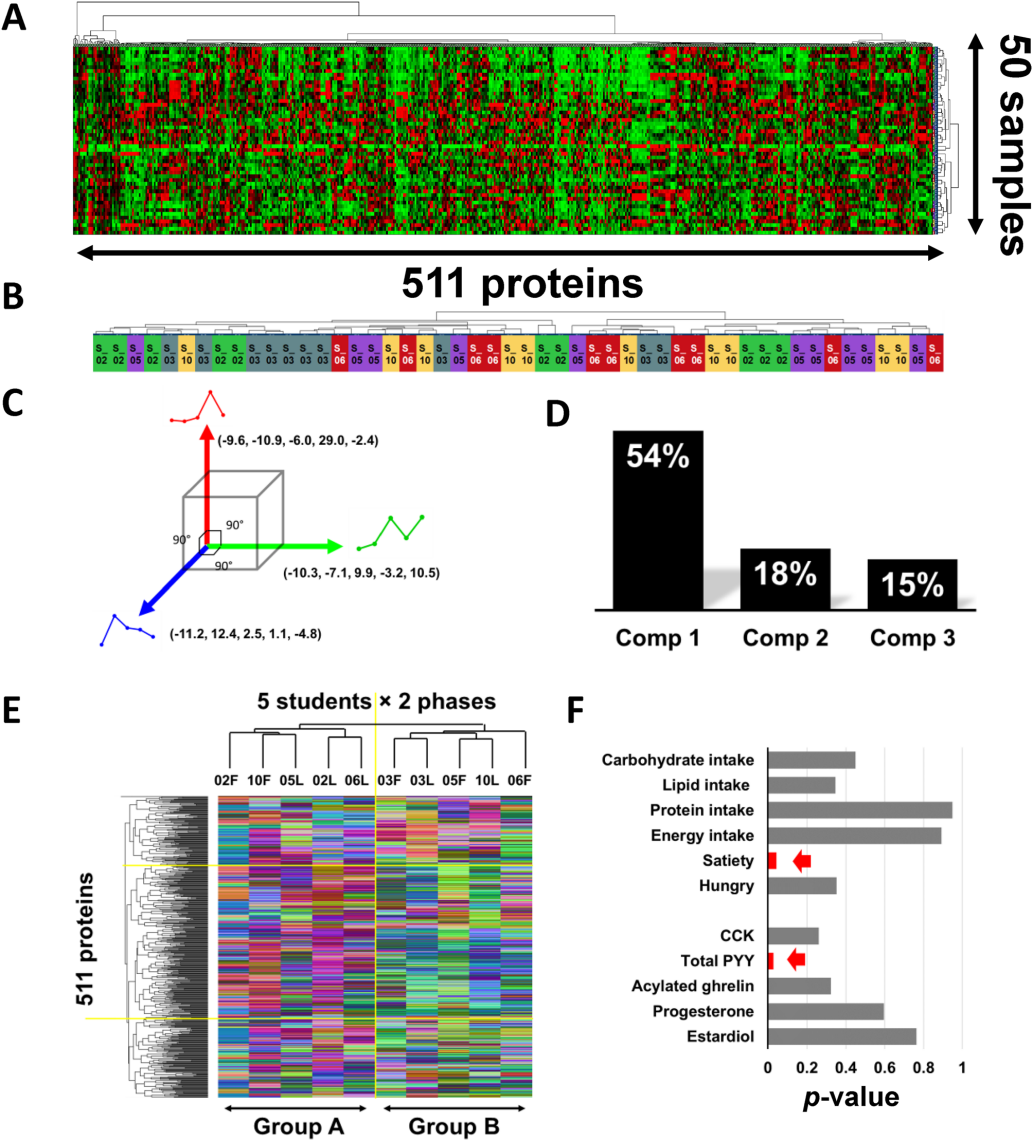
Heatmap analysis and transition pattern classification. (**A**) Heatmap analysis depicting the expression levels of 511 proteins across 50 samples: red: up-regulated, green: down-regulated. Proteins and samples were categorized by cluster analysis. (**B**) Three orthogonal transition bases obtained by PCA analysis. (**C**) Contributions of each principal components to the original data. (**D**) Transitional pattern heatmap with cluster analysis; Each protein transition pattern during exercise dissolved into three basic patterns, and the cosines to the three bases were used for categorization. Cosines were further converted to RGB colors to visualize the categorization.

The original Article has been corrected.

