# Supplementary figures and images for: Proteomics of appetite-regulating system influenced by menstrual cycle and intensive exercise in female athletes: a pilot study
Source: Sci Rep. 2024 Feb 20;14:4188. doi: 10.1038/s41598-024-54572-1 (PMC10879539; doi:10.1038/s41598-024-54572-1)

|     | 0 min                                                                              | 30 min                                                                             | 60 min                                                                             | 105 min                                                                             | 150 min                                                                              |
|-----|------------------------------------------------------------------------------------|------------------------------------------------------------------------------------|------------------------------------------------------------------------------------|-------------------------------------------------------------------------------------|--------------------------------------------------------------------------------------|
| S02 | 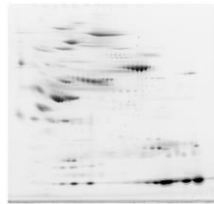  | 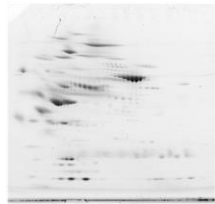  | 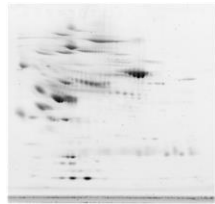  | 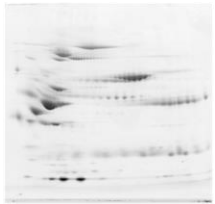  | 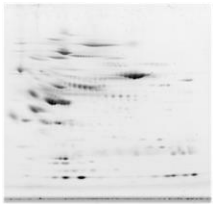  |
| S03 | 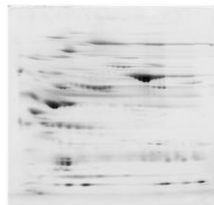  | 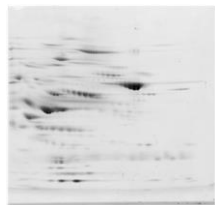  | 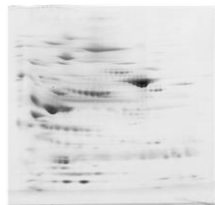  | 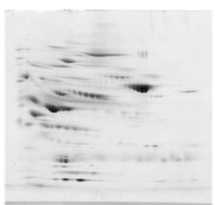  | 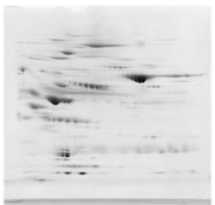  |
| S05 | 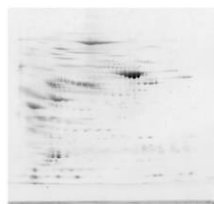  | 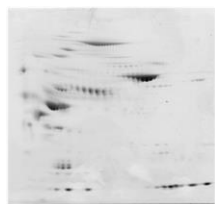  | 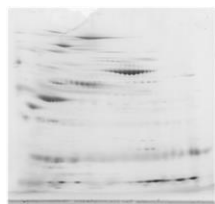  | 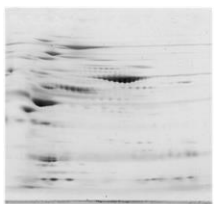  | 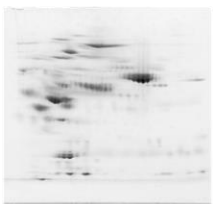  |
| S06 | 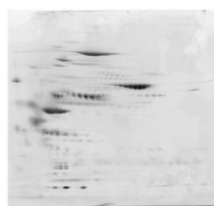  | 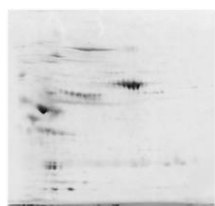  | 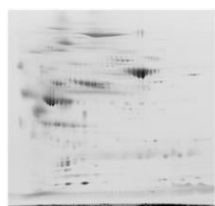  | 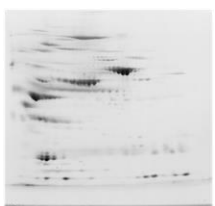  | 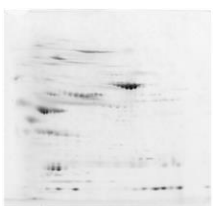  |
| S10 | 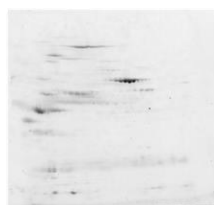 | 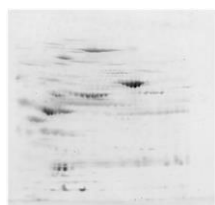 | 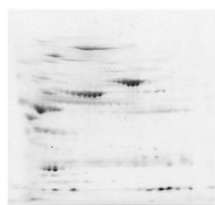 | 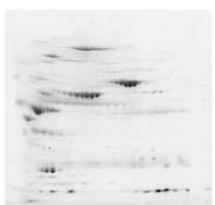 | 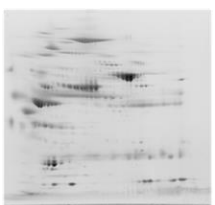 |

**Supplementary Figure 1   Gel images in follicular phase**

Supplement: Supplementary file 1 — Supplementary Figure 1. [file 41598_2024_54572_MOESM1_ESM.pdf]

|            | 0 min                                                                               | 30 min                                                                              | 60 min                                                                              | 105 min                                                                              | 150 min                                                                               |
|------------|-------------------------------------------------------------------------------------|-------------------------------------------------------------------------------------|-------------------------------------------------------------------------------------|--------------------------------------------------------------------------------------|---------------------------------------------------------------------------------------|
| <b>S02</b> | 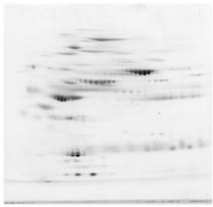   | 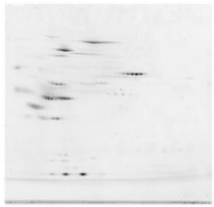   | 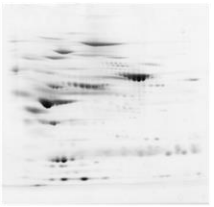   | 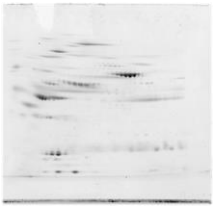   | 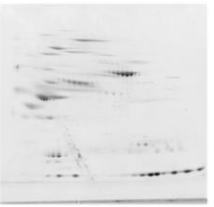   |
| <b>S03</b> | 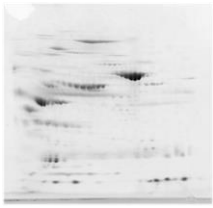   | 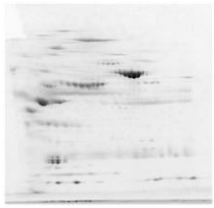   | 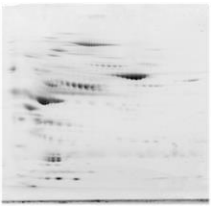   | 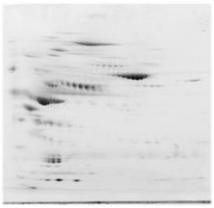   | 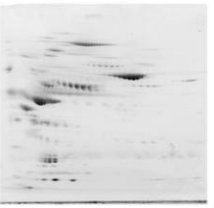   |
| <b>S05</b> | 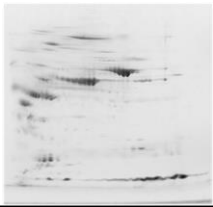   | 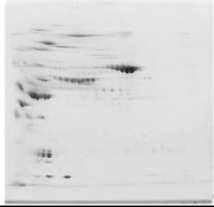   | 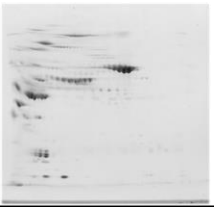   | 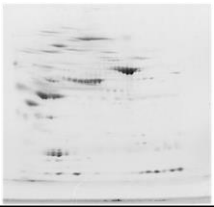   | 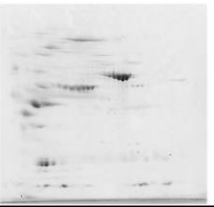   |
| <b>S06</b> | 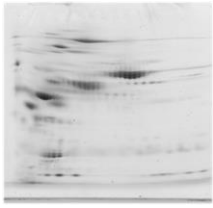   | 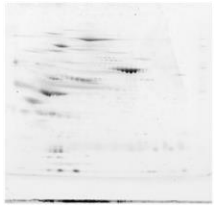   | 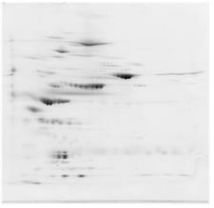   | 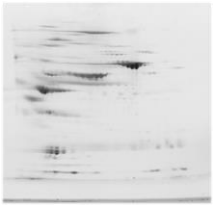   | 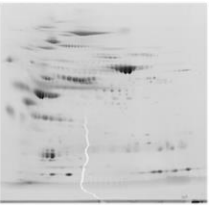   |
| <b>S10</b> | 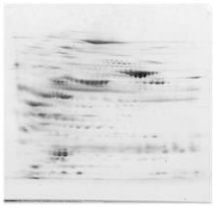 | 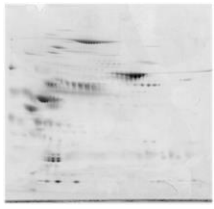 | 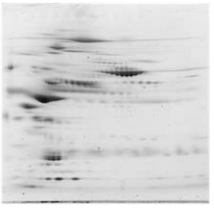 | 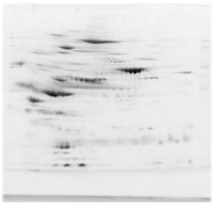 | 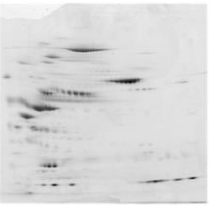 |

**Supplementary Figure 2      Gel images   in luteal phase**

Supplement: Supplementary file 2 — Supplementary Figure 2. [file 41598_2024_54572_MOESM2_ESM.pdf]

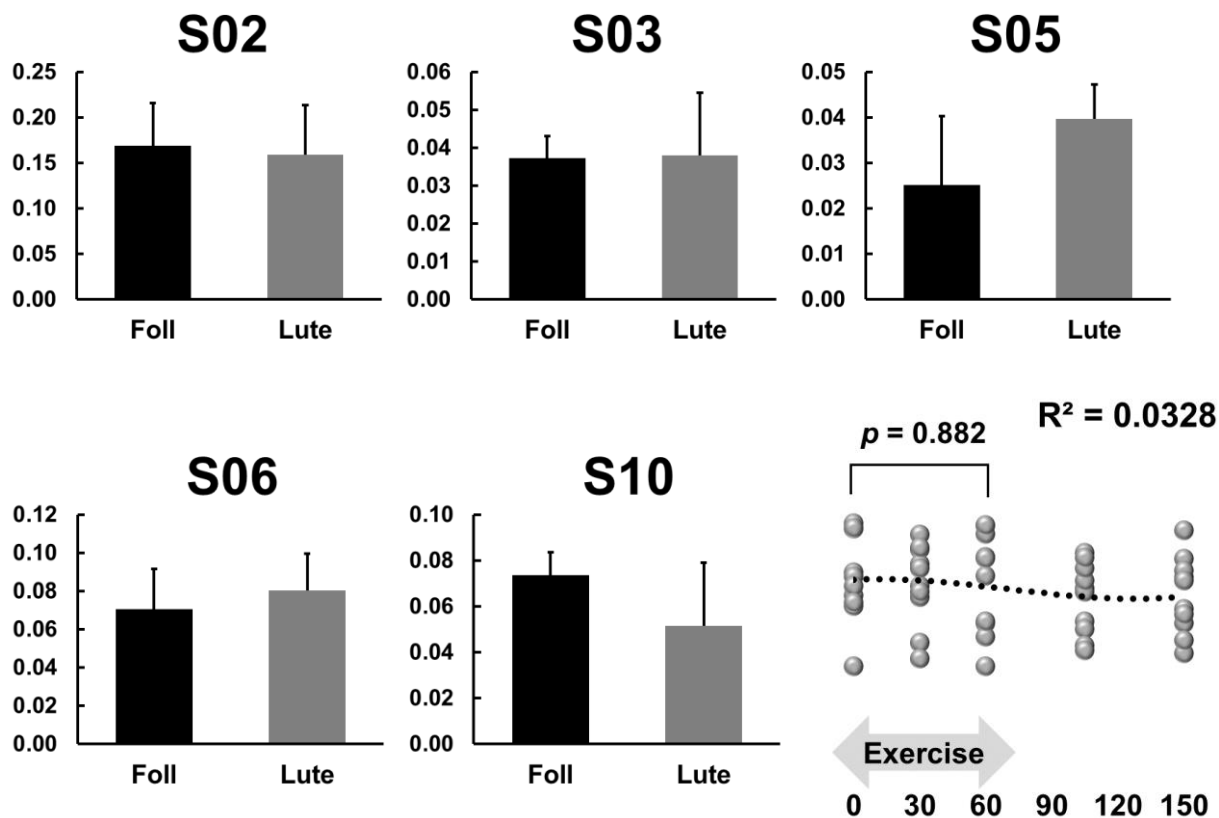

Supplementary Figure 3

Supplement: Supplementary file 3 — Supplementary Figure 3. [file 41598_2024_54572_MOESM3_ESM.pdf]

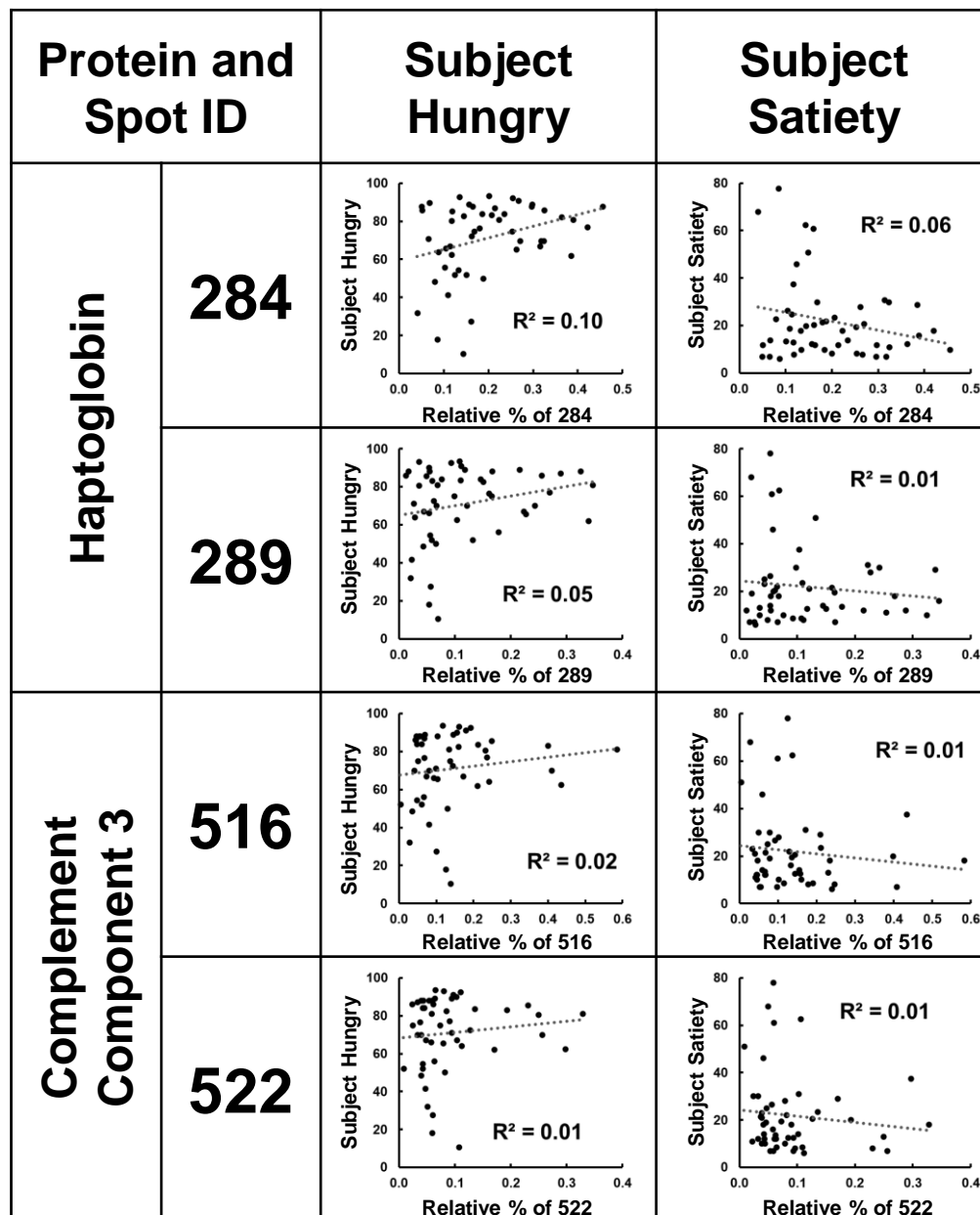

**Supplementary Figure 4**

Supplement: Supplementary file 4 — Supplementary Figure 4. [file 41598_2024_54572_MOESM4_ESM.pdf]

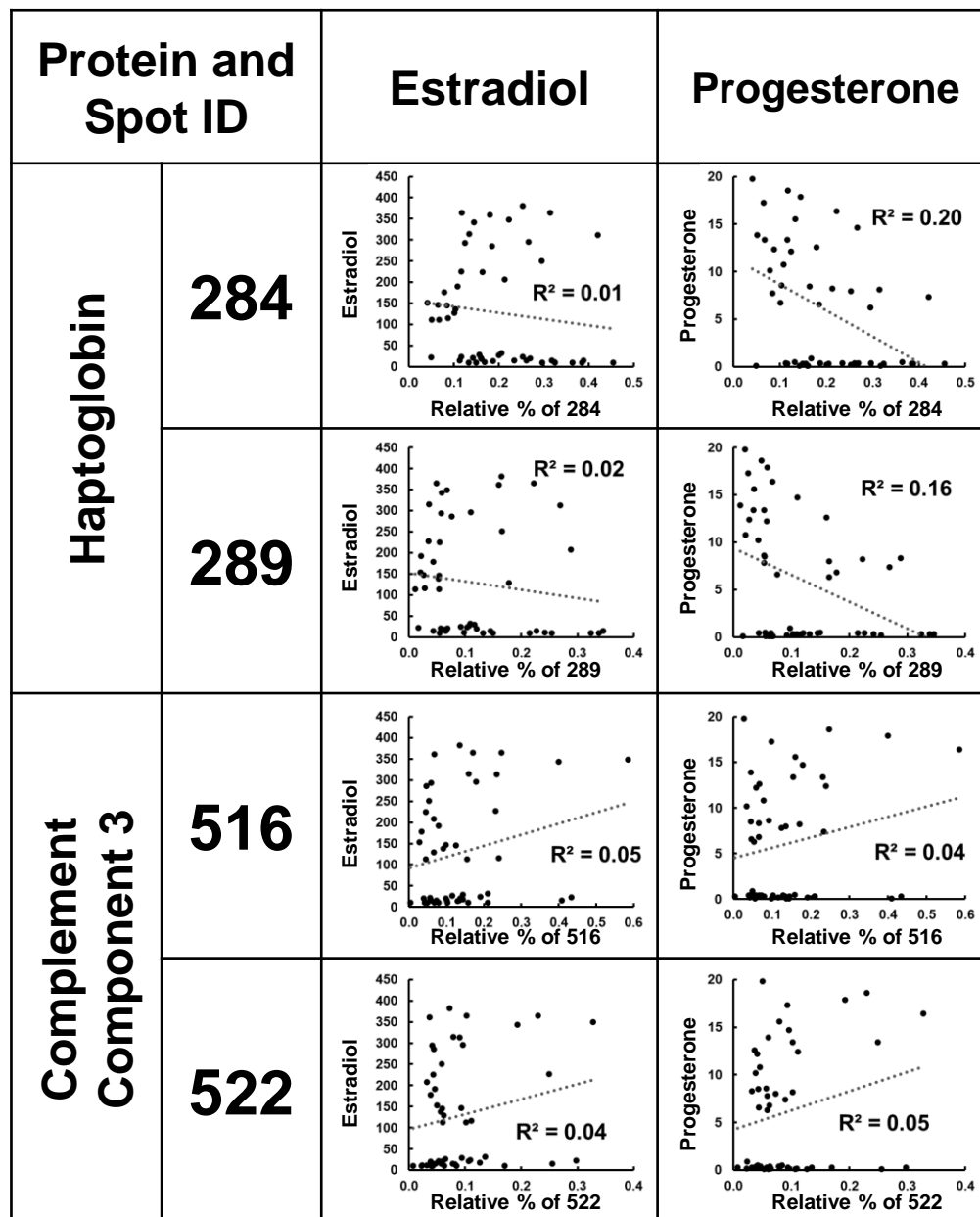

**Supplementary Figure 5**

Supplement: Supplementary file 5 — Supplementary Figure 5. [file 41598_2024_54572_MOESM5_ESM.pdf]

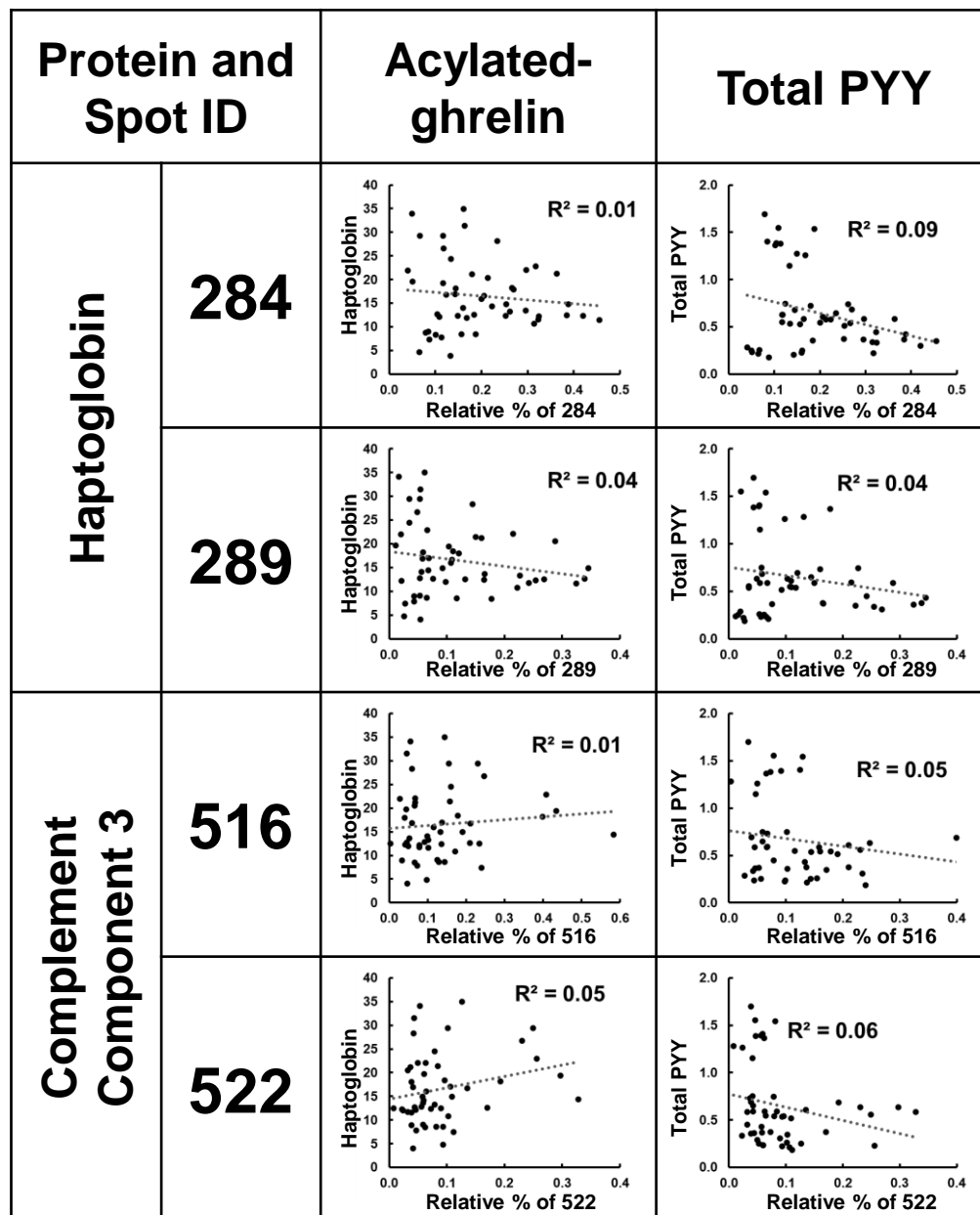

**Supplementary Figure 6**

Supplement: Supplementary file 6 — Supplementary Figure 6. [file 41598_2024_54572_MOESM6_ESM.pdf]
