# Supplementary material for: Proteomics of appetite-regulating system influenced by menstrual cycle and intensive exercise in female athletes: a pilot study
Source: Sci Rep. 2024 Feb 20;14:4188. doi: 10.1038/s41598-024-54572-1 (PMC10879539; doi:10.1038/s41598-024-54572-1)
Supplement: Supplementary file 7 — Supplementary Legends. [file 41598_2024_54572_MOESM7_ESM.docx]

Supplementary Figure 1: 2DE gel images depicting serum proteins during the follicular phase.

Supplementary Figure 2: 2DE gel images depicting serum proteins during the luteal phase.

Supplementary Figure 3: Changes in serum IgM levels during the menstrual cycle and exercise. Foll: Follicular phase, Lute: Luteal phase.

Supplementary Figure 4: Correlation between subjective hunger and satiety and haptoglobin and complement component 3.

Supplementary Figure 5: Correlation between estradiol and progesterone and haptoglobin and complement component 3.

Supplementary Figure 6: Correlation between acylated-ghrelin, PYY, and haptoglobin and complement component 3.
